# Serial Explantation After Parathyroid Autotransplant for Recurrent Hypercalcemia in Chronic Renal Failure

**DOI:** 10.5812/ijem.14102

**Published:** 2014-04-01

**Authors:** William Robert Foley, Dara Lundon, Yvonnne O’Meara, Tom Gorey

**Affiliations:** 1 School of Medicine and Medical Sciences, University College Dublin, Dublin, Ireland; 2 Department of Nephrology, Mater Misericordiae University Hospital, Dublin, Ireland; 3 General, Breast and Endocrine Surgery, Mater Misericordiae University Hospital, Dublin, Ireland

**Keywords:** Parathyroid hormone, Hyperparathyroidism, Hypercalcemia

## Abstract

**Introduction:**

Development of autonomous parathyroid gland function can occur in cases of long standing renal disease, leading to hyperparathyroidism and hypercalcemia. Debate exists over the optimum surgical treatment strategy and the choice lies with the individual surgeon. We illustrated the method of total parathyroidectomy and autotransplantation to the forearm and proposed it to be superior to both total and subtotal parathyroidectomy.

**Case Presentation:**

This case illustrated the development of secondary and subsequently tertiary hyperparathyroidism in a 66-year-old man with a history of chronic renal failure. The patient was managed surgically by parathyroid autotransplantation and serial explantation.

**Discussion:**

Refractory hypercalcemia due to autonomous parathyroid tissue following parathyroidectomy can be managed with greater ease and efficacy by serial explantation of autotransplanted tissue versus a more difficult re-exploration of the neck.

## 1. Introduction

Hyperparathyroidism is caused by an excess of parathyroid hormone either from a pathological change in function within the parathyroid gland (primary and tertiary hyperparathyroidism) or from an appropriate physiological response to low calcium levels (secondary hyperparathyroidism). Primary hyperparathyroidism is a condition with an incidence of 22 per 100,000 of the adult population (1). The incidence increases with age, and reaches its peak between the ages of 50 and 60 years. The disease occurs in a female to male ratio of 3:1. A single benign adenoma accounts for 89% of primary hyperparathyroidism with a double adenoma accounting for just fewer than 5%; the remainder are due to diffuse hyperplasia in all four parathyroid glands (2). Secondary hyperparathyroidism is an adaptive process in which an extrinsic abnormal change affecting calcium homeostasis stimulates production of parathyroid hormone (3). It is very common in end stage renal disease (ESRD) and especially in those under dialysis. These patients are hypocalcemic with raised parathyroid hormone levels.

Tertiary hyperparathyroidism develops in patients with long standing secondary hyperparathyroidism. Those who do not receive therapy for hypocalcemia or those whose parathyroid hormone cannot be suppressed, develop autonomous parathyroid function and hypercalcemia. It is characterized by high serum levels of both calcium and parathyroid hormone. The high levels of parathyroid hormone in these diseases lead to osteolytic bone destruction and renal osteodystrophy whilst hypercalcemia can cause confusion, nephrolithiasis, and GI disturbances. Parathyroid glands exhibit diffuse hyperplasia throughout all four glands in 71% of patients, with nodular hyperplasia accounting for 26% and a single adenoma found in only 3% (4), very much in contrast to the number of glands affected in primary hyperparathyroidism. The aim of this report is to illustrate the most effective management of refractory hyperparathyroidism in chronic renal failure.

## 2. Case Presentation

**Figure 1.**
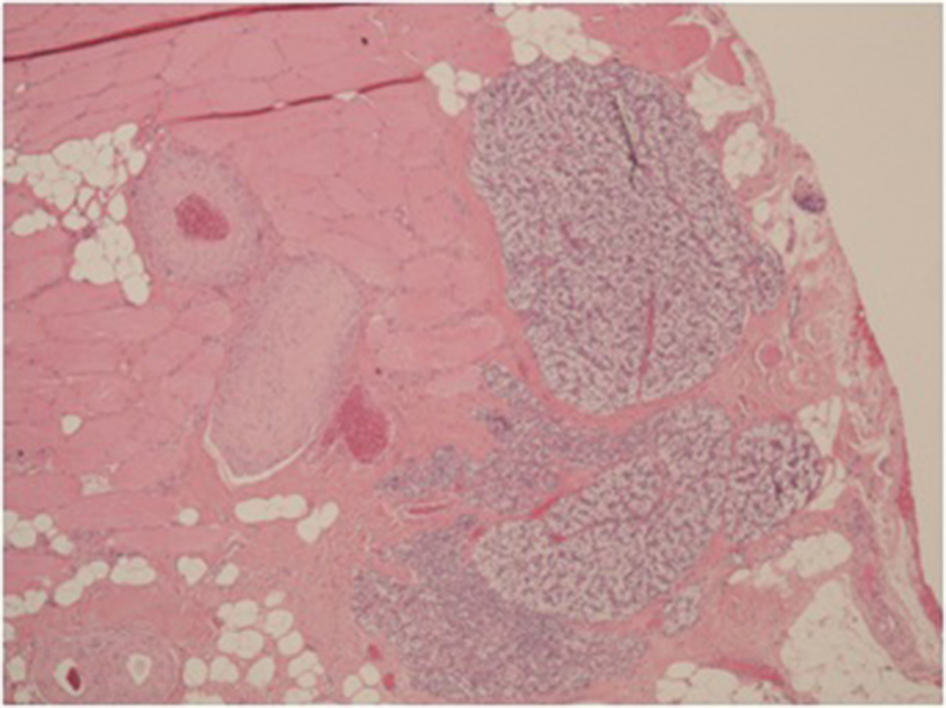
Explant; Focal Areas of Parathyroid Tissue Within Muscle Tissue

We report a 66-year-old man with a history of ESRD secondary to IgA nephropathy. The patient had first received a renal transplant in 2007. In 2008, a technetium 99m-labeled sestamibi scintigraphy scan of the parathyroid was obtained due to persistently elevated serum parathyroid hormone levels with normocalcemia. The scan demonstrated diffuse uptake in each of the four glands. The patient was diagnosed with secondary hyperparathyroidism and medical treatment with Cinacalcet and oral calcium supplements was initiated. The patient was admitted in September 2009 under the care of the renal service with symptomatic uremia and hyperkalemia. His serum parathyroid hormone levels were also elevated to 125 pmol/L, over 16-times the upper limit of normal (reference range 1.9-7.6). Medical treatment was continued, however, due to refractory parathyroid hormone levels, surgical management was deemed necessary. In March 2010, an open parathyroidectomy with autotransplantation of one-half of a parathyroid gland in three muscle pockets of the forearm was undertaken; surgical clips were used in the procedure to mark the re-implanted tissue. The histology of the remaining tissue illustrated four-gland hyperplasia. Serum parathyroid hormone decreased to 45 pmol/L four days postoperatively. The patient’s parathyroid hormone and calcium levels were stabilized over the following 29 months. Following cadaveric kidney transplant in 2012, the patient’s parathyroid hormone elevated to 236.5 pmol/L and he was subsequently scheduled for explantation of one section of autotransplanted parathyroid tissue. Technetium 99m-labeled sestamibi scintigraphy demonstrated no uptake in the neck or mediastinal area and no uptake in the forearm. Therefore under local anaesthesia, the most distal forearm section of parathyroid tissue was located and removed. Histology confirmed the presence of parathyroid tissue hyperplasia; see Figure 1. The serum parathyroid hormone level fell to 27.9 pmol/L 13 days following the explantation. A spike in the levels to 265 pmol/L consistent with refractory hyperparathyroidism was noted 34 days after the procedure. Consequently, a second section of previously autotransplanted parathyroid tissue was excised and the final section marked with a nonabsorbable suture to aid in its localization if a third explant being deemed necessary.

**Figure 2.**
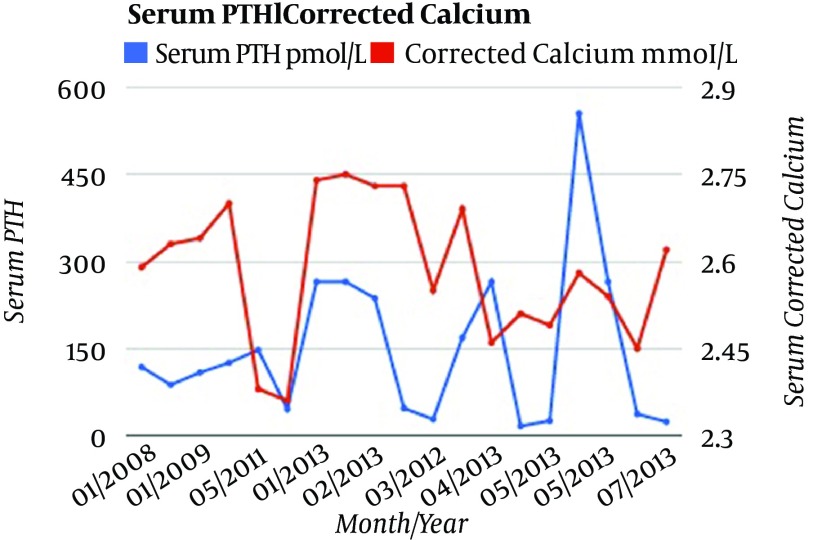
Corrected Calcium and Parathyroid Hormone Levels Illustrating Refractory Hyperparathyroidism, Following the Original Autotransplant and Following each Explantation

The parathyroid levels then fell to their lowest recorded level of 16.2 pmol/L; however, in the face of the levels rising within 1 month (to 555 pmol/L), a third explantation was planned. However parathyroid hormone levels subsequently fell after 18 days and on July 2013, the serum parathyroid level was found to be 23.6 pmol/L; Figure 2. 

This patient's serum calcium and parathyroid would be monitored to assess the need for a third and final explant. Then, our patient would be expected to become hypocalcemic and would be treated medically for this. However, if parathyroid levels were to remain elevated, then the possibility of an ectopic focus of parathyroid tissue would be considered.

## 3. Discussion

**Figure 3.**
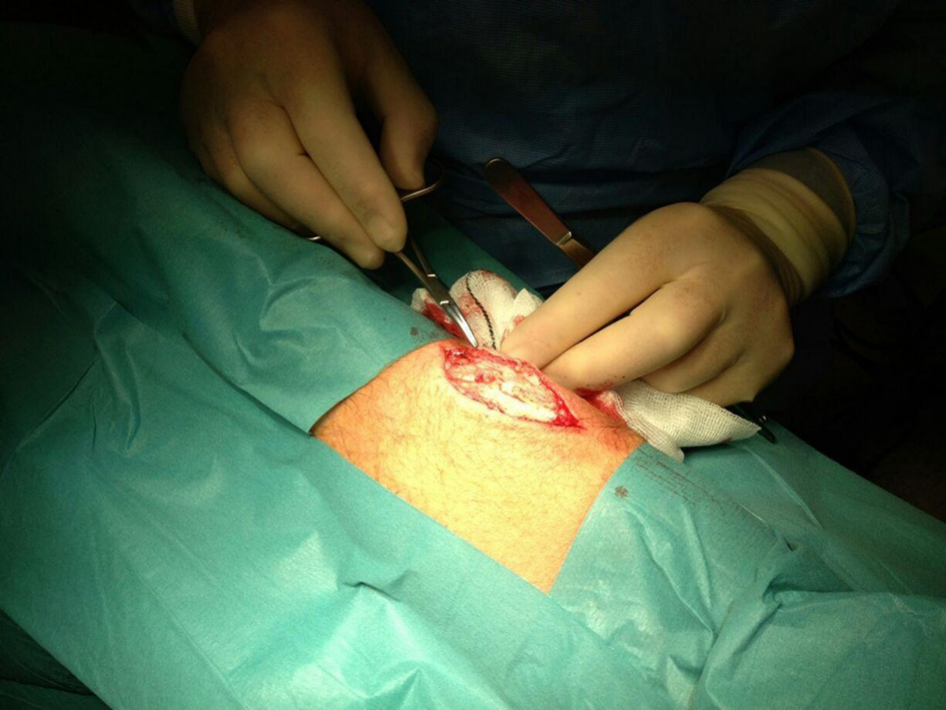
Location of Autotransplant in the Forearm

The options for management of secondary hyperparathyroidism include prophylactic measures such as oral calcium and calcitriol supplementation. Management options for patients refractory to these treatments include calcimimetics, such as Cinacalcet (5), phosphate binders, vitamin D analogues (6), and surgery. The surgical options for management of refractory hyperparathyroidism are total parathyroidectomy with autotransplantation (7) of one half of one gland, usually in the forearm (Figure 3), subtotal parathyroidectomy (removal of three and a half glands), and total parathyroidectomy without autotransplantation. Parathyroidectomy and autotransplantation to the forearm in patients with chronic renal failure offers a chance for stabilizing serum parathyroid hormone and calcium levels and reducing the risk of hypocalcaemia. In cases of recurrent hyperparathyroidism after initial parathyroidectomy, refractory parathyroid tissue can easily be removed under local anesthesia to negate the need for the more difficult re-exploration of the neck, as would be the case in subtotal parathyroidectomy (8). Serial explantation can be used to manage refractory hyperparathyroidism until serum parathyroid hormone levels normalize. Imaging of hyperplastic parathyroid tissue after forearm autotransplantation can be performed using technetium 99m-labeled sestamibi scintigraphy. This technique is effective in localizing overactive parathyroid tissue in 77% of patients with primary hyperparathyroidism (9). Eighty-one percent of solitary adenomas can be localized, however, poor sensitivity (37%) would be seen if more than one gland is involved (9). In fact, a poor uptake is a predictor of multiple gland disease in primary hyperparathyroidism (10). Many reports of technetium 99m-labeled sestamibi scintigraphy employment to localize the hyperfunctioning parathyroid tissue after autotransplantation are from case studies (11-13). It has been reported, that uptake in recurrent hyperparathyroidism after autotransplantation with technetium 99m-labeled sestamibi scintigraphy is over 95% (14, 15). However, the correct value is most likely lower than this in reality, as the majority of transplanted tissue show diffuse hyperplasia (4) and this is associated with a reduced uptake with technetium 99m-labeled sestamibi scintigraphy (9). Confounders could include the small sample sizes used to get to this data and possible publication bias. A study with a larger sample size is necessary in order to ascertain a more reliable value for efficacy of using technetium 99m-labeled sestamibi scintigraphy to localize hyperfunctioning parathyroid tissue in cases of autotransplantation.

Total parathyroidectomy and autotransplantation to the forearm is a superior choice to subtotal parathyroidectomy. The former method allows for easier management of refractory disease and negates the need for both general anesthesia and surgical re-exploration of the neck. However, there has been no randomized control trial comparing these existing approaches; therefore, the advantages and disadvantages of each approach should be considered in each individual patient to make the right decision.

## References

[1] Adami S, Marcocci C, Gatti D (2002). Epidemiology of primary hyperparathyroidism in Europe.. J Bone Miner Res..

[2] Fraker DL, Harsono H, Lewis R (2009). Minimally invasive parathyroidectomy: benefits and requirements of localization, diagnosis, and intraoperative PTH monitoring. long-term results.. World J Surg..

[3] Fraser WD (2009). Hyperparathyroidism.. Lancet..

[4] Kebebew E, Duh QY, Clark OH (2004). Tertiary hyperparathyroidism: histologic patterns of disease and results of parathyroidectomy.. Arch Surg..

[5] Block GA, Martin KJ, de Francisco AL, Turner SA, Avram MM, Suranyi MG (2004). Cinacalcet for secondary hyperparathyroidism in patients receiving hemodialysis.. N Engl J Med..

[6] Duranton F, Rodriguez-Ortiz ME, Duny Y, Rodriguez M, Daures JP, Argiles A (2013). Vitamin D treatment and mortality in chronic kidney disease: a systematic review and meta-analysis.. Am J Nephrol..

[7] Wells SA, Jr., Gunnells JC, Shelburne JD, Schneider AB, Sherwood LM (1975). Transplantation of the parathyroid glands in man: clinical indications and results.. Surgery..

[8] Rothmund M, Wagner PK, Schark C (1991). Subtotal parathyroidectomy versus total parathyroidectomy and autotransplantation in secondary hyperparathyroidism: a randomized trial.. World J Surg..

[9] McHenry CR, Lee K, Saadey J, Neumann DR, Esselstyn CB, Jr. (1996). Parathyroid localization with technetium-99m-sestamibi: a prospective evaluation.. J Am Coll Surg..

[10] Sebag F, Hubbard JG, Maweja S, Misso C, Tardivet L, Henry JF (2003). Negative preoperative localization studies are highly predictive of multiglandular disease in sporadic primary hyperparathyroidism.. Surgery..

[11] Sippel RS, Bianco J, Chen H (2003). Radioguided parathyroidectomy for recurrent hyperparathyroidism caused by forearm graft hyperplasia.. J Bone Miner Res..

[12] Cutress RI, Manwaring-White C, Dixon K, Dhir A, Skene AI (2009). Gamma probe radioguided parathyroid forearm surgery in recurrent hyperparathyroidism.. Ann R Coll Surg Engl..

[13] Ardito G, Revelli L, Giustozzi E, Giordano A (2012). Radioguided parathyroidectomy in forearm graft for recurrent hyperparathyroidism.. Br J Radiol..

[14] Hindie E, Zanotti-Fregonara P, Just PA, Sarfati E, Melliere D, Toubert ME (2010). Parathyroid scintigraphy findings in chronic kidney disease patients with recurrent hyperparathyroidism.. Eur J Nucl Med Mol Imaging..

[15] Itoh K, Ishizuka R (2003). Tc-99m-MIBI scintigraphy for recurrent hyperparathyroidism after total parathyroidectomy with autograft.. Ann Nucl Med..

